# A theoretical model of temperate phages as mediators of gut microbiome dysbiosis

**DOI:** 10.12688/f1000research.18480.1

**Published:** 2019-07-01

**Authors:** Derek M. Lin, Henry C. Lin

**Affiliations:** 1 Medicine Service, New Mexico VA Health Care System, Albuquerque, New Mexico, USA; 2 Department of Gastroenterology and Hepatology, University of New Mexico, Albuquerque, New Mexico, USA

**Keywords:** Bacteriophage, phage, gut, microbiome, virome, western diet, prophage-encoded genes, dysbiosis, fecal microbiota transplant

## Abstract

Bacteriophages are the most prominent members of the gut microbiome, outnumbering their bacterial hosts by a factor of 10. Phages are bacteria-specific viruses that are gaining attention as highly influential regulators of the gut bacterial community. Dysregulation of the gut bacterial community contributes to dysbiosis, a microbiome disorder characterized by compositional and functional changes that contribute to disease. A role for phages in gut microbiome dysbiosis is emerging with evidence that the gut phage community is altered in dysbiosis-associated disorders such as colorectal cancer and inflammatory bowel disease. Several recent studies have linked successful fecal microbiota transplantation to uptake of the donor’s gut phage community, offering some insight into why some recipients respond to treatment whereas others do not. Here, we review the literature supporting a role for phages in mediating the gut bacterial community, giving special attention to Western diet dysbiosis as a case study to demonstrate a theoretical phage-based mechanism for the establishment and maintenance of dysbiosis.

## Introduction

Insights into the relationship between diet and the gut microbiome have significantly advanced our understanding of nutrition as a mediator of health and disease. The influence of the Western diet specifically on dysbiosis in the gut bacterial community is well established ^1^. Conspicuously lacking from this body of research is a fundamental understanding of the gut virome, sometimes referred to as the “dark matter” of the microbiome ^2^. It has long been known that the virome harbors genes for antibiotic resistance and bacterial toxins ^3, 4^, contributing to the virulence of clinically relevant bacterial pathogens ^5, 6^. Emerging evidence implicates an altered gut virome in colorectal cancer, inflammatory bowel disease, and other states associated with microbiome dysbiosis ^7– 10^. The role of the virome, whether as cause or consequence of dysbiosis, is unclear.

## Gut microbiome dysbiosis

Gut microbiome dysbiosis broadly encompasses the various states of perturbed gut microbial community composition associated with disease or disorder in the host. In a healthy gut environment, the resident (commensal) gut bacteria (that is, symbionts) are non-pathogenic. A healthy gut microbiota–host interaction is a mutualism in which gut bacteria thrive in the gastrointestinal environment of the host while providing the host with multiple benefits through metabolism, immune system development, and protection from pathogens ^11^. During dysbiosis, the homeostatic balance of this symbiosis shifts, disrupting the beneficial nature of the relationship and contributing to disease states. In this setting, otherwise beneficial gut symbionts may transition to a state of pathogenicity (that is, pathobionts). Dysbiosis is implicated in the etiology of numerous clinical disorders ranging from those involving the digestive tract, such as inflammatory bowel disease and non-alcoholic steatohepatitis, to those outside the digestive tract, such as atherosclerotic cardiovascular disease and autism ^12– 15^.

## The gut virome

Evidence on the role of gut viruses during dysbiosis is limited. The predominant members of the gut virome are bacteria-specific viruses called bacteriophages (phages). Phages are most commonly associated with phage therapy, a practice of administering lytic phages to control bacterial pathogens (for example, *Staphylococcus aureus*, *Escherichia coli*, and *Pseudomonas aeruginosa*) ^16^. About 10 ^14^ viruses, comprised of about 1200 virotypes, reside in the gut ^17^; this population is 10 times as abundant as gut bacteria but comparable in diversity ^18, 19^. Contemporary studies of the gut bacterial community rely on next-generation sequencing of the universal 16s rRNA bacterial gene, which provides a compositional readout of the microbiome. This approach is not possible with phages as they lack a conserved phylogenetic marker ^20^, one of many challenges in phage research. Even the gold standard for studying phage communities, viral metagenomics, is limited by the lack of a genomic library to compare against the enormous diversity of uncharacterized phage genes collectively referred to as “viral dark matter” ^2^.

**Figure 1.**
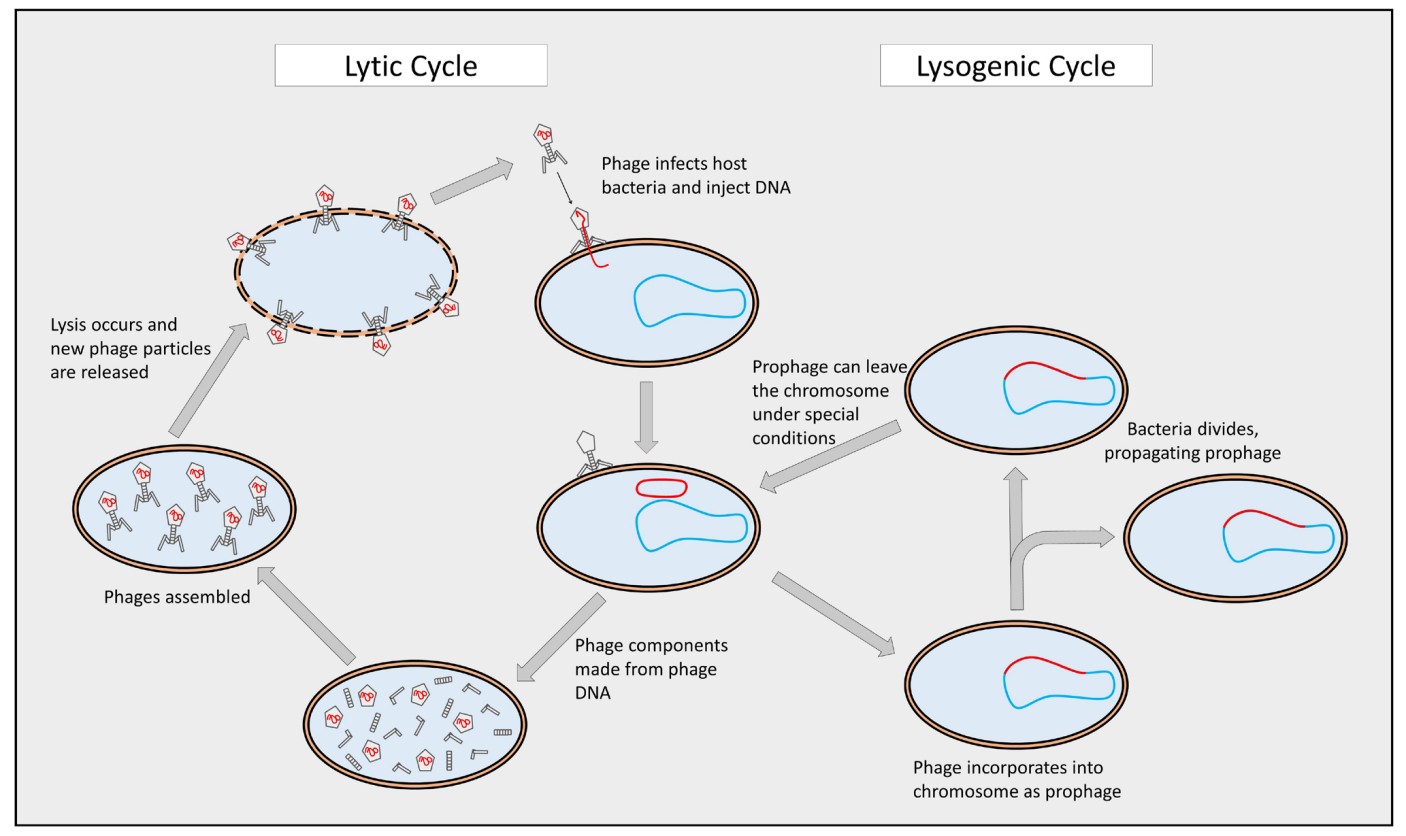
Reproductive life cycles of a temperate phage. Temperate phages can reproduce via both lytic and lysogenic cycles. The decision as to which cycle gets induced depends on environmental factors. This simplified version of phage life cycles demonstrates how the cycles are intertwined.

Advances in gut phage research have demonstrated a role for phages as agents of compositional change in the gut microbial community ^21, 22^. Phages exert enormous evolutionary pressure on microbial communities by lysing their bacterial hosts or by mediating gene transfer ^23^. In oceanic surface waters, phage-mediated lysis leads to the death of 20 to 40% of the total bacterial population every 24 hours ^24^. In the gut, phages are primarily temperate and able to incorporate into the bacterial chromosome as latent prophages, thereby reproducing with the bacterial host (that is, the lysogenic cycle); prophages then may produce phage progeny by inducing the lytic cycle ( Figure 1) ^20^. Temperate phages also have the option of reproducing by entering the lytic cycle directly and immediately lysing their bacterial host. About half of all sequenced bacterial genomes in the GenBank database contain at least one prophage, and some species can harbor up to 15 ^25^. Prophages are intimately linked to bacterial resilience and function, encoding genes for metabolism, antibacterial resistance, and toxin production (for example, shiga toxin production), thereby providing fitness benefits for the bacterial host ^6, 9^. Prophages further enhance fitness in their bacterial hosts by preventing infection from other phages (that is, superinfection exclusion) and by lysing competing bacteria (that is, “kill-the-relative”) ^26, 27^. Some phage-encoded genes are required for commensal gut bacteria to form a mutualism with their host ^28^; conversely, phage-encoded virulence factors promote pathogenic behavior in their host ^6, 29^. These findings are highly relevant to the study of gut microbiome dysbiosis, which can be characterized by the transition of commensal bacteria from symbiont to pathobiont. The transition to dysbiosis is multifactorial and once established it becomes difficult to treat ^30^.

## A role for phages in mediating dysbiosis

Dysbiosis is associated with an increased abundance and richness of the mucosal temperate phage population ^7– 9, 31, 32^. Prophages in the gut are spontaneously induced into the lytic cycle at a modest baseline level ^33^. Large-scale induction typically requires environmental stressors that cause a community-wide “SOS response” in the bacterial hosts ^34^. The SOS response is triggered by DNA damage in the bacterial chromosome ^35^, essentially signaling to the prophage that the bacterial host is no longer suitable. The prophage reacts by inducing the lytic cycle to produce a new population of phage progeny, which seek out new hosts to infect. The inflamed gut is associated with an upregulated SOS response in resident gut bacteria ^36^, loss of phage diversity ^32^, and elevated levels prophage induction ^37^. Elevated prophage induction is a mechanism of horizontal gene transfer between bacterial hosts, increasing rates of genetic recombination and diversification of phage-encoded genes ^38^. This process has been found to drive the evolution of bacterial pathogens by expanding the reservoir of phage-encoded genes for virulence factors and antibiotic resistance ^5, 37^. In the setting of an infection by enteric pathogens such as shiga toxin–producing *E. coli*, antibiotics that trigger the SOS response activate shiga toxin synthesis through the phage induction pathway; this can lead to diarrhea, hemorrhagic colitis, hemolytic-uremic syndrome, and even death ^6^. Prophages encode virulence factors for other clinically relevant pathogens, including *Vibrio cholerae*, *S. aureus*, *Corynebacterium diphtheriae*, and *Clostridium botulinum* ^29^ *.* In addition to directly encoding genes for toxins, phage genes can indirectly upregulate production of bacterial toxins and can influence adhesion, colonization, and invasion ^39^.

Within the gut environment, phages are suspected of mediating diversification of the non-pathogenic, commensal microbial community ^40^. Temperate phages are theorized to act on the commensal microbiota via “community shuffling”, whereby prophage induction in response to SOS-triggering stressors may increase the pathobiont-to-symbiont ratio observed during dysbiosis ^41^. Lending support to this theory is the finding that changes to gut phage community composition precede the onset of type 1 diabetes in children ^42^. Furthermore, recovery from *Clostridium difficile* dysbiosis after fecal microbiota transplantation (FMT) is associated with an uptake of the donor phage community ^43– 45^. Collectively, there is strong evidence of a role for gut phages in numerous disease states associated with gut microbiome dysbiosis.

## Western diet dysbiosis as a case study

**Figure 2.**
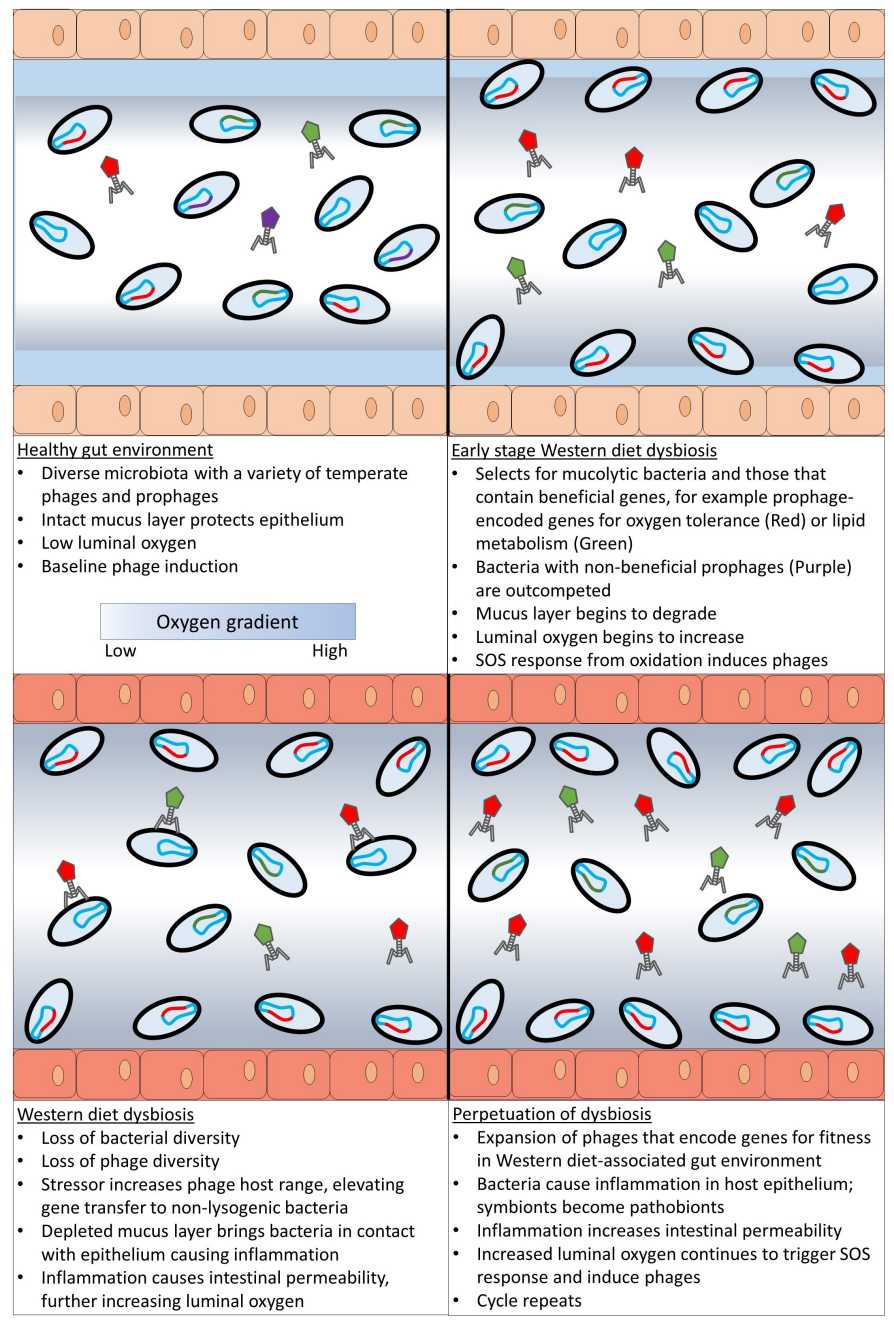
Theoretical model for phage-mediated dysbiosis. Prophages can drive otherwise commensal bacterial hosts (symbionts) to behave pathogenically (pathobionts) when exposed to environmental stressors, such as those associated with a Western diet. Phage-encoded genes support bacterial mechanisms for bacterial survival at the cost of the human host. The pathogenic behavior of these resident gut microbes promotes inflammation in the intestinal epithelium, which perpetuates the state of environmental stress and persistent dysbiosis.

A Western diet, characterized by a high-fat, high-sugar, and low-fiber intake, is one of the most clinically important disruptors of the gut microbiome. The substantial body of literature on the Western diet provides an ideal case study for this theoretical model, illustrating the potential role of phages in mediating dysbiosis (a graphical representation of this model can be found in Figure 2). It should be noted that a “Western” diet is often generalized as “high fat” when it has been shown that the type of fat is an important factor in the onset of dysbiosis and disease ^46, 47^. Indeed, a Western diet can be classified differently but is commonly associated with high levels of either n-6 polyunsaturated fats or saturated fats ^1^.

In a Western diet–induced mouse model of obesity, adverse effects include glucose intolerance and fatty liver, both of which improve after treatment with norfloxacin and ampicillin ^48^, demonstrating the role of an antibiotic-sensitive microbiome. Antibiotic treatment reverses the state of increased intestinal permeability, a common outcome of a Western diet that results in elevated plasma concentration of bacterial endotoxins ^49^. These findings suggest that a Western diet can disrupt homeostatic balance in the gut microbiome, promoting a state of increased intestinal permeability, increased endotoxin absorption, and endotoxemia. The endotoxin, in turn, drives the immune response of the host, thereby producing inflammation, glucose intolerance, and the other characteristic features of metabolic syndrome.

A Western diet is distinguished by not only its high lipid content but also its low availability of fiber. Deprived of fiber from the diet, the gut microbiota compensates by foraging host glycans from the epithelial mucus layer ^50^. Whether phages mediate genes related to glycan foraging has yet to be studied. A healthy mucus layer is a protective boundary separating the sensitive host epithelium from the pro-inflammatory contents of the intestinal lumen (for example, bacterial endotoxins). Degradation of the mucus layer by glycan-foraging bacteria depletes this protection, bringing luminal bacteria closer to the epithelium, thus promoting inflammation and increased intestinal permeability ^51, 52^. A more permeable intestinal lining is hypothesized to allow more oxygen from the bloodstream to enter the normally anoxic intestinal lumen ^53^, inflicting oxidative stress on obligate anaerobes in the gut ^54^. This idea is supported by the finding that oxygen respiration becomes a dominant metabolic signature in mouse models of colitis ^55^. Using the phosphorescence quenching method, Albenberg *et al*. (2014) showed that there is an oxygen gradient radially in the gut lumen whereby the oxygen concentration is highest near the mucosa ^56^. Accordingly, the composition of the gut bacterial community is organized radially, and mucosally associated bacteria are the most oxygen-tolerant ^57^. It is now appreciated that the intestinal barrier does not simply have two states, impermeable and permeable, but rather there exists degrees of permeability. However, there is a consensus that increased permeability associated with bacterial translocation and immune activation is harmful and leads to chronic inflammation ^11, 53^. A change in oxygen concentration may also affect the gut phage population as oxidative stress is a trigger of the SOS response and can induce prophages to enter the lytic cycle. In support of this hypothesis is a study by Kim and Bae (2016), who demonstrated that a Western diet expands phage-encoded genes for oxidative tolerance ^9^. It is unlikely that oxidative stress on commensal bacteria is solely responsible for inducing gut phages, as the metabolic by-products of a Western diet have also been shown to trigger prophage induction ^58^.

A Western diet has been found to increase the population density of phages in the gut mucosa and expand the reservoir of phage-encoded genes for phage reproduction ^9^. Higher rates of temperate phage infection can cause pathogenicity in commensal gut bacteria by disrupting the function of bacterial genes ^29^. Systematic disruption of bacterial genes by phages may have the potential to drive the entire gut microbial community toward a state of increased pathogenic potential (that is, dysbiosis). Whether other disruptors of the gut microbiome—including antibiotic therapy, inflammation, exposure to anesthesia, surgery or other traumas, immune deficiency, and exposure to toxins—exert their effects on the microbiome via its phage population remains to be tested.

Most laboratory observations suggest that phages generally have a narrow host range (all bacteria a phage can infect) ^23^, although recent evidence suggests that phages have a much broader host range in the gut ** than what has been observed *in vitro* ^59, 60^. Stressors that induce prophages (for example, antibiotic treatment) consequently broaden phage host range and expand the reservoir of phage-encoded genes for surviving the stressor (for example, antibiotic resistance genes) ^5^. Diet is a potential regulator of prophage induction among commensal gut bacteria ^59^, and unregulated prophage induction may be responsible for disturbing the homeostatic relationship between microbiome and host. Since perturbation of the gut environment by a Western diet upregulates phage-encoded genes for lipid metabolism and oxidative tolerance ^9^, it can be reasonably postulated that these phages likely confer a competitive advantage to their bacterial hosts in a gut environment with higher availability of lipids and increased levels of luminal oxygen. Phages with the combination of broad host range and genes for bacterial host fitness may have a substantial competitive advantage in the gut microbial community, thereby propagating these genes across taxonomically distinct bacterial species within the microbiota and increasing competition for limited resources such as host glycans from the mucus layer. The microbiome composition during Western diet dysbiosis shifts with an expansion of bacteria in the phyla *Proteobacteria* and *Firmicutes* ^61^; *Proteobacteria* specifically are associated with numerous dysbioses, intestinal barrier dysfunction, and low-grade inflammation ^57^. The majority of gut prophages are found in the genomes of these two phyla ^59^, and there is a positive correlation between viral content and *Firmicutes* in the feces during obesity ^62^.

There is clear evidence that prophages can help make the decision for their bacterial hosts to act as either symbionts or pathobionts ^6, 28^ and consumption of a Western diet may influence that decision. Outlined above is one possible mechanism for the evolution of a phage community that promotes pathogen-like behavior in otherwise commensal gut bacteria through elevated prophage induction, wherein bacterial survival may come at a cost to the human host. This theoretical model also takes into account the loss of phage diversity observed in response to gut microbiome perturbations, which may be responsible for the persistence of dysbiosis and resistance to clinical treatment. It is worth noting that our proposed model is not mutually exclusive with other mechanisms that may lead to Western diet–associated dysbiosis, such as those involving host metabolism in response to specific dietary fats ^47^ or host genetic factors ^63, 64^.

## Moving forward

As demonstrated by Howe *et al*. (2016), a Western diet mouse model reduces both bacterial and phage diversity ^65^. After mice are transitioned back to a standard diet, diversity of the bacterial community returns to pre–Western diet levels whereas the diversity of the phage community remains low. It is possible that dietary disturbances permanently alter phage diversity as well as the functional nature of the phage-encoded gene reservoir; the long-term impacts of this lost diversity are unknown. Treatment of dysbiosis may be contingent on re-establishing a healthy phage community.

Recent developments in clinical FMT therapy have identified an association between successful treatment of *C. difficile* infection and uptake of the donor’s fecal phage community by the FMT recipient ^43, 45^. A clinical study found that administration of a bacteria-depleted fecal filtrate (phages retained) was sufficient for treatment of *C. difficile* infection and transition of the recipient phage community toward that of the donor ^44^. In an ulcerative colitis mouse model, FMT responders had reduced populations of mucosal phages compared with non-responders ^31^. The composition of the donor’s phage community has yet to be considered an important factor for FMT. For that matter, the recipient’s diet has also not been considered. Gut phages heavily influence the composition of the gut microbiota and in turn its relationship with the human host. Much research still needs to be carried out to determine how the phage community responds to perturbation and contributes to the establishment, maintenance, and remediation of dysbiosis.

## Abbreviation

FMT, fecal microbiota transplantation

## References

[1] Martinez KB Leone V Chang EB: Western diets, gut dysbiosis, and metabolic diseases: Are they linked? *Gut Microbes.* 2017;8(2):130–42. 10.1080/19490976.2016.1270811 28059614 PMC5390820

[2] Shkoporov AN Hill C: Bacteriophages of the Human Gut: The "Known Unknown" of the Microbiome. *Cell Host Microbe.* 2019;25(2):195–209. 10.1016/j.chom.2019.01.017 30763534

[3] Berg DE Davies J Allet B: Transposition of R factor genes to bacteriophage lambda. *Proc Natl Acad Sci U S A.* 1975;72(9):3628–32. 10.1073/pnas.72.9.3628 1059152 PMC433049

[4] Smith HW Green P Parsell Z: Vero cell toxins in Escherichia coli and related bacteria: transfer by phage and conjugation and toxic action in laboratory animals, chickens and pigs. *J Gen Microbiol.* 1983;129(10):3121–37. 10.1099/00221287-129-10-3121 6418852

[5] Modi SR Lee HH Spina CS: Antibiotic treatment expands the resistance reservoir and ecological network of the phage metagenome. *Nature.* 2013;499(7457):219–22. 10.1038/nature12212 23748443 PMC3710538

[6] Zhang X McDaniel AD Wolf LE: Quinolone antibiotics induce Shiga toxin-encoding bacteriophages, toxin production, and death in mice. *J Infect Dis.* 2000;181(2):664–70. 10.1086/315239 10669353

[7] Norman JM Handley SA Baldridge MT: Disease-specific alterations in the enteric virome in inflammatory bowel disease. *Cell.* 2015;160(3):447–60. 10.1016/j.cell.2015.01.002 25619688 PMC4312520

[8] Lepage P Colombet J Marteau P: Dysbiosis in inflammatory bowel disease: a role for bacteriophages? *Gut.* 2008;57(3):424–5. 10.1136/gut.2007.134668 18268057

[9] Kim MS Bae JW: Spatial disturbances in altered mucosal and luminal gut viromes of diet-induced obese mice. *Environ Microbiol.* 2016;18(5):1498–510. 10.1111/1462-2920.13182 26690305

[10] Nakatsu G Zhou H Wu WKK: Alterations in Enteric Virome Are Associated With Colorectal Cancer and Survival Outcomes. *Gastroenterology.* 2018;155(2):529–541.e5. 10.1053/j.gastro.2018.04.018 29689266

[11] DeGruttola AK Low D Mizoguchi A: Current Understanding of Dysbiosis in Disease in Human and Animal Models. *Inflamm Bowel Dis.* 2016;22(5):1137–50. 10.1097/MIB.0000000000000750 27070911 PMC4838534

[12] Tamboli CP Neut C Desreumaux P: Dysbiosis in inflammatory bowel disease. *Gut.* 2004;53(1):1–4. 10.1136/gut.53.1.1 14684564 PMC1773911

[13] Henao-Mejia J Elinav E Jin C: Inflammasome-mediated dysbiosis regulates progression of NAFLD and obesity. *Nature.* 2012;482(7384):179–85. 10.1038/nature10809 22297845 PMC3276682

[14] Serino M Blasco-Baque V Nicolas S: Far from the eyes, close to the heart: dysbiosis of gut microbiota and cardiovascular consequences. *Curr Cardiol Rep.* 2014;16(11):540. 10.1007/s11886-014-0540-1 25303894 PMC4194023

[15] Williams BL Hornig M Buie T: Impaired carbohydrate digestion and transport and mucosal dysbiosis in the intestines of children with autism and gastrointestinal disturbances. *PLoS One.* 2011;6(9):e24585. 10.1371/journal.pone.0024585 21949732 PMC3174969

[16] Lin DM Koskella B Lin HC: Phage therapy: An alternative to antibiotics in the age of multi-drug resistance. *World J Gastrointest Pharmacol Ther.* 2017;8(3):162–173. 10.4292/wjgpt.v8.i3.162 28828194 PMC5547374

[17] Breitbart M Hewson I Felts B: Metagenomic analyses of an uncultured viral community from human feces. *J Bacteriol.* 2003;185(20):6220–3. 10.1128/jb.185.20.6220-6223.2003 14526037 PMC225035

[18] Sender R Fuchs S Milo R: Revised Estimates for the Number of Human and Bacteria Cells in the Body. *PLoS Biol.* 2016;14(8):e1002533. 10.1371/journal.pbio.1002533 27541692 PMC4991899

[19] Lozupone CA Stombaugh JI Gordon JI: Diversity, stability and resilience of the human gut microbiota. *Nature.* 2012;489(7415):220–30. 10.1038/nature11550 22972295 PMC3577372

[20] Reyes A Semenkovich NP Whiteson K: Going viral: next-generation sequencing applied to phage populations in the human gut. *Nat Rev Micro.* 2012;10(9):607–17. 10.1038/nrmicro2853 22864264 PMC3596094

[21] Bao HD Pang MD Olaniran A: Alterations in the diversity and composition of mice gut microbiota by lytic or temperate gut phage treatment. *Appl Microbiol Biotechnol.* 2018;102(23):10219–30. 10.1007/s00253-018-9378-6 30302521

[22] Hsu BB Gibson TE Yeliseyev V: Bacteriophages dynamically modulate the gut microbiota and metabolome. *bioRxiv.* 2018 10.1101/454579

[23] Koskella B Brockhurst MA: Bacteria-phage coevolution as a driver of ecological and evolutionary processes in microbial communities. *FEMS Microbiol Rev.* 2014;38(5):916–31. 10.1111/1574-6976.12072 24617569 PMC4257071

[24] Suttle CA: The significance of viruses to mortality in aquatic microbial communities. *Microb Ecol.* 1994;28(2):237–43. 10.1007/BF00166813 24186450

[25] Touchon M Bernheim A Rocha EP: Genetic and life-history traits associated with the distribution of prophages in bacteria. *ISME J.* 2016;10(11):2744–54. 10.1038/ismej.2016.47 27015004 PMC5113838

[26] De Paepe M Leclerc M Tinsley CR: Bacteriophages: an underestimated role in human and animal health? *Front Cell Infect Microbiol.* 2014;4:39. 10.3389/fcimb.2014.00039 24734220 PMC3975094

[27] Bondy-Denomy J Qian J Westra ER: Prophages mediate defense against phage infection through diverse mechanisms. *ISME J.* 2016;10(12):2854–66. 10.1038/ismej.2016.79 27258950 PMC5148200

[28] Oliver KM Degnan PH Hunter MS: Bacteriophages encode factors required for protection in a symbiotic mutualism. *Science.* 2009;325(5943):992–4. 10.1126/science.1174463 19696350 PMC5473335

[29] Brüssow H Canchaya C Hardt WD: Phages and the evolution of bacterial pathogens: from genomic rearrangements to lysogenic conversion. *Microbiol Mol Biol Rev.* 2004;68(3):560–602, table of contents. 10.1128/MMBR.68.3.560-602.2004 15353570 PMC515249

[30] Weiss GA Hennet T: Mechanisms and consequences of intestinal dysbiosis. *Cell Mol Life Sci.* 2017;74(16):2959–77. 10.1007/s00018-017-2509-x 28352996 PMC11107543

[31] Gogokhia L Buhrke K Bell R: Expansion of Bacteriophages Is Linked to Aggravated Intestinal Inflammation and Colitis. *Cell Host Microbe.* 2019;25(2):285–299.e8. 10.1016/j.chom.2019.01.008 30763538 PMC6885004

[32] Zuo T Lu XJ Zhang Y: Gut mucosal virome alterations in ulcerative colitis. *Gut.* 2019;68(7):1169–1179. 10.1136/gutjnl-2018-318131 30842211 PMC6582748

[33] Reyes A Wu M McNulty NP: Gnotobiotic mouse model of phage-bacterial host dynamics in the human gut. *Proc Natl Acad Sci U S A.* 2013;110(50):20236–41. 10.1073/pnas.1319470110 24259713 PMC3864308

[34] Allen HK Looft T Bayles DO: Antibiotics in feed induce prophages in swine fecal microbiomes. *mBio.* 2011;2(6): pii: e00260-11. 10.1128/mBio.00260-11 22128350 PMC3225969

[35] Dwyer DJ Kohanski MA Collins JJ: Role of reactive oxygen species in antibiotic action and resistance. *Curr Opin Microbiol.* 2009;12(5):482–9. 10.1016/j.mib.2009.06.018 19647477 PMC2761529

[36] Butala M Zgur-Bertok D Busby SJ: The bacterial LexA transcriptional repressor. *Cell Mol Life Sci.* 2009;66(1):82–93. 10.1007/s00018-008-8378-6 18726173 PMC11131485

[37] Diard M Bakkeren E Cornuault JK: Inflammation boosts bacteriophage transfer between *Salmonella* spp. *Science.* 2017;355(6330):1211–5. 10.1126/science.aaf8451 28302859

[38] Touchon M Moura de Sousa JA Rocha EP: Embracing the enemy: the diversification of microbial gene repertoires by phage-mediated horizontal gene transfer. *Curr Opin Microbiol.* 2017;38:66–73. 10.1016/j.mib.2017.04.010 28527384

[39] Wagner PL Waldor MK: Bacteriophage control of bacterial virulence. *Infect Immun.* 2002;70(8):3985–93. 10.1128/iai.70.8.3985-3993.2002 12117903 PMC128183

[40] Scanlan PD: Bacteria-Bacteriophage Coevolution in the Human Gut: Implications for Microbial Diversity and Functionality. *Trends Microbiol.* 2017;25(8):614–23. 10.1016/j.tim.2017.02.012 28342597

[41] Mills S Shanahan F Stanton C: Movers and shakers: influence of bacteriophages in shaping the mammalian gut microbiota. *Gut Microbes.* 2013;4(1):4–16. 10.4161/gmic.22371 23022738 PMC3555884

[42] Zhao G Vatanen T Droit L: Intestinal virome changes precede autoimmunity in type I diabetes-susceptible children. *Proc Natl Acad Sci U S A.* 2017;114(30):E6166–E6175. 10.1073/pnas.1706359114 28696303 PMC5544325

[43] Chehoud C Dryga A Hwang Y: Transfer of Viral Communities between Human Individuals during Fecal Microbiota Transplantation. *mBio.* 2016;7(2):e00322. 10.1128/mBio.00322-16 27025251 PMC4817255

[44] Ott SJ Waetzig GH Rehman A: Efficacy of Sterile Fecal Filtrate Transfer for Treating Patients With *Clostridium difficile* Infection. *Gastroenterology.* 2017;152(4):799–811.e7. 10.1053/j.gastro.2016.11.010 27866880

[45] Zuo T Wong SH Lam K: Bacteriophage transfer during faecal microbiota transplantation in *Clostridium difficile* infection is associated with treatment outcome. *Gut.* 2018;67(4):634–43. 10.1136/gutjnl-2017-313952 28539351 PMC5868238

[46] Huang EY Leone VA Devkota S: Composition of dietary fat source shapes gut microbiota architecture and alters host inflammatory mediators in mouse adipose tissue. *JPEN J Parenter Enteral Nutr.* 2013;37(6):746–54. 10.1177/0148607113486931 23639897 PMC3812400

[47] Devkota S Wang Y Musch MW: Dietary-fat-induced taurocholic acid promotes pathobiont expansion and colitis in Il10-/- mice. *Nature.* 2012;487(7405):104–8. 10.1038/nature11225 22722865 PMC3393783

[48] Membrez M Blancher F Jaquet M: Gut microbiota modulation with norfloxacin and ampicillin enhances glucose tolerance in mice. *FASEB J.* 2008;22(7):2416–26. 10.1096/fj.07-102723 18326786

[49] Cani PD Bibiloni R Knauf C: Changes in gut microbiota control metabolic endotoxemia-induced inflammation in high-fat diet-induced obesity and diabetes in mice. *Diabetes.* 2008;57(6):1470–81. 10.2337/db07-1403 18305141

[50] Desai MS Seekatz AM Koropatkin NM: A Dietary Fiber-Deprived Gut Microbiota Degrades the Colonic Mucus Barrier and Enhances Pathogen Susceptibility. *Cell.* 2016;167(5):1339–1353.e21. 10.1016/j.cell.2016.10.043 27863247 PMC5131798

[51] Kim YS Ho SB: Intestinal goblet cells and mucins in health and disease: recent insights and progress. *Curr Gastroenterol Rep.* 2010;12(5):319–30. 10.1007/s11894-010-0131-2 20703838 PMC2933006

[52] Johansson ME Gustafsson JK Sjöberg KE: Bacteria penetrate the inner mucus layer before inflammation in the dextran sulfate colitis model. *PLoS One.* 2010;5(8):e12238. 10.1371/journal.pone.0012238 20805871 PMC2923597

[53] Rigottier-Gois L: Dysbiosis in inflammatory bowel diseases: the oxygen hypothesis. *ISME J.* 2013;7(7):1256–61. 10.1038/ismej.2013.80 23677008 PMC3695303

[54] Zeng MY Inohara N Nuñez G: Mechanisms of inflammation-driven bacterial dysbiosis in the gut. *Mucosal Immunol.* 2017;10(1):18–26. 10.1038/mi.2016.75 27554295 PMC5788567

[55] Hughes ER Winter MG Duerkop BA: Microbial Respiration and Formate Oxidation as Metabolic Signatures of Inflammation-Associated Dysbiosis. *Cell Host Microbe.* 2017;21(2):208–19. 10.1016/j.chom.2017.01.005 28182951 PMC5313043

[56] Albenberg L Esipova TV Judge CP: Correlation between intraluminal oxygen gradient and radial partitioning of intestinal microbiota. *Gastroenterology.* 2014;147(5):1055–63.e8. 10.1053/j.gastro.2014.07.020 25046162 PMC4252572

[57] Litvak Y Byndloss MX Tsolis RM: Dysbiotic *Proteobacteria* expansion: a microbial signature of epithelial dysfunction. *Curr Opin Microbiol.* 2017;39:1–6. 10.1016/j.mib.2017.07.003 28783509

[58] Oh JH Alexander LM Pan M: Dietary Fructose and Microbiota-Derived Short-Chain Fatty Acids Promote Bacteriophage Production in the Gut Symbiont *Lactobacillus reuteri*. *Cell Host Microbe.* 2019;25(2):273–284.e6. 10.1016/j.chom.2018.11.016 30658906

[59] Kim MS Bae JW: Lysogeny is prevalent and widely distributed in the murine gut microbiota. *ISME J.* 2018;12(4):1127–41. 10.1038/s41396-018-0061-9 29416123 PMC5864201

[60] Ogilvie LA Jones BV: The human gut virome: a multifaceted majority. *Front Microbiol.* 2015;6:918. 10.3389/fmicb.2015.00918 26441861 PMC4566309

[61] Hildebrandt MA Hoffmann C Sherrill-Mix SA: High-fat diet determines the composition of the murine gut microbiome independently of obesity. *Gastroenterology.* 2009;137(5):1716–1724.e2. 10.1053/j.gastro.2009.08.042 19706296 PMC2770164

[62] Yadav H Jain S Nagpal R: Increased fecal viral content associated with obesity in mice. *World J Diabetes.* 2016;7(15):316–20. 10.4239/wjd.v7.i15.316 27555892 PMC4980638

[63] Kostic AD Gevers D Siljander H: The dynamics of the human infant gut microbiome in development and in progression toward type 1 diabetes. *Cell Host Microbe.* 2015;17(2):260–73. 10.1016/j.chom.2015.01.001 25662751 PMC4689191

[64] Knights D Silverberg MS Weersma RK: Complex host genetics influence the microbiome in inflammatory bowel disease. *Genome Med.* 2014;6(12):107. 10.1186/s13073-014-0107-1 25587358 PMC4292994

[65] Howe A Ringus DL Williams RJ: Divergent responses of viral and bacterial communities in the gut microbiome to dietary disturbances in mice. *ISME J.* 2016;10(5):1217–27. 10.1038/ismej.2015.183 26473721 PMC5029215

